# Bridging cognition and control through passive eye movement integration in motor imagery brain-computer interfaces

**DOI:** 10.3389/fnhum.2026.1849674

**Published:** 2026-06-02

**Authors:** Alessio D’Aquino, Thomas Schack

**Affiliations:** 1 Neurocognition and Action Biomechanics Group, Faculty of Psychology and Sports Science, Bielefeld University, Bielefeld, Germany; 2 Center for Cognitive Interaction Technology (CITEC), Bielefeld University, Bielefeld, Germany; 3 Research Institute for Cognition and Robotics (Cor-Lab), Bielefeld University, Bielefeld, Germany

**Keywords:** brain-computer interface, cognitive state, eye movements, hybrid BCI, motor imagery, neurorehabilitation, passive monitoring, visuomotor integration

## Abstract

Motor Imagery (MI) Brain-Computer Interfaces (BCIs) represent a promising technology for neurorehabilitation and assistive control. However, the clinical viability of these systems is frequently hindered by the inherent limitations of electroencephalography (EEG) with regard to its low signal-to-noise ratio (SNR), non-stationarity, and high inter-subject variability. Standard decoding methods often fail to capture the complexity of user intention leading to unreliable performance and user frustration. This review proposes a solution to these challenges by advocating for the integration of passive eye movements (EM) as a complementary data stream. The theoretical rationale for this approach rests on the neurocognitive principle of functional equivalence. Because imagined actions recruit similar visuomotor networks to those used in physical execution, EM constitute a robust correlate of the underlying neural simulation. We distinguish this approach from conventional hybrid systems that use gaze coordinates for active control. Instead, we argue for a framework of passive monitoring where oculomotor metrics, including pupil diameter, fixation patterns, and saccadic dynamics, serve as a continuous window into the user’s cognitive state. We synthesize evidence demonstrating that these passive signals can reliably index cognitive load, attentional allocation, and covert motor planning. By fusing these behavioral metrics with EEG, a BCI can disambiguate uncertain neural patterns and verify user intent without imposing additional task demands. Furthermore, we discuss how this multimodal integration enables the development of adaptive classifiers that respond to fluctuations in user fatigue and engagement. Bridging the gap between cognition and control through passive EM monitoring offers a pathway to create BCI systems that are intrinsically responsive to the user’s internal state.

## 1 Introduction

Brain-Computer Interfaces (BCIs) have attracted considerable attention in recent years especially in the areas of neurorehabilitation and assistive technologies (Belkhiria et al., 2022; Wolpaw et al., 2020). By converting neural information into commands for external devices, BCIs introduce novel methods of communication and control for both able-bodied individuals and people with severe motor impairments thereby functioning as an effective tool for restoring interaction and promoting autonomy (Müller-Putz et al., 2016; Saibene et al., 2023; Yi et al., 2024). Conventional BCI interfaces translate user intentions into actions such as selecting an item on a screen or controlling a robotic arm (Ang and Guan, 2017; Collinger et al., 2013). Here user intention is understood as the conscious action or goal the user intends to execute through the interface. This is commonly achieved by measuring brain activity often using conventional non-invasive modalities like electroencephalography (EEG) or functional near-infrared spectroscopy though invasive methods also exist. Regardless of the modality, the central goal is to capture user intention and translate it into device control. Despite such broad applications, considerable challenges persist in how well BCIs encapsulate the complexity and richness of user intentions especially in real-world scenarios (Niazi et al., 2011; Xu et al., 2014). Techniques predominantly acquire and process one signal at a time (e.g., EEG) often resulting in inaccuracies due to signal contamination from artifacts, such as electromyography (EMG) or electrooculography (EOG), along with high inter-individual variability in brain signals and task proficiency including BCI illiteracy in some users (Kilicarslan and Contreras-Vidal, 2019; Saha and Baumert, 2020). Further complications arise from non-stationary fluctuations in user states like attention or fatigue (Alzahab et al., 2021). The limitation of single-signal processing is particularly apparent in motor imagery (MI) paradigms where the user’s communication and control over an external interface occur by mentally rehearsing an action without physically moving. Although ocular artifacts frequently contaminate EEG readings, eye movements (EM) offer valuable insights into a user’s focus, attentional state, and intentions, assuming they are appropriately utilized.

Fusing multiple data streams is a key insight that suggests a paradigm shift toward hybrid BCIs with the aim of improving accuracy and robustness (Kim et al., 2015; Millán et al., 2010; Zhang et al., 2019). The logic for this integration rests on the growing body of evidence that perception, MI, and action share overlapping neural substrates (Fuchs and Belardinelli, 2021). This cardinal principle of motor cognition states that imagining an action is a process that recruits similar visuomotor networks used during physical execution leading to observable neural as well as oculomotor correlates (Grèzes and Decety, 2001; Jeannerod, 1994; Moran et al., 2012; Ruiz et al., 2024). Indeed, principles of visuomotor coordination appear largely preserved when actions are imagined demonstrating that imagery engages both sensory and motor processes (D'Aquino et al., 2023; McCormick et al., 2013). A hybrid BCI can leverage the intrinsic synergy between the neural and oculomotor correlates of imagined action thus enabling it to benefit from the complementary strengths of both modalities. Whereas EEG grants direct access to cortical activity with high temporal resolution and affords insights into the immediate neural dynamics of MI (McFarland and Wolpaw, 2011), EM present physiologically distinct signals that act as reliable indices of attentional allocation, cognitive load, and motor planning (Eckstein et al., 2017; Henderson, 2003; Hebb, 1968). These behavioral metrics have the additional advantage of being less susceptible to certain types of noise and delivering robust indicators of spatial intent (Dimigen and Ehinger, 2021; Holmqvist et al., 2011). Therefore, the combination of EM with EEG-based control may yield significant benefits including reducing ambiguity and enhancing interface responsiveness (Jacob, 1995; Shishkin et al., 2016). Such a prospect is especially relevant in neurorehabilitation where assimilating gaze signals can transform a BCI from marginally operable to fully serviceable for individuals with severe motor impairments (Leeb et al., 2013; Wen et al., 2021).

Although the promise of hybrid BCIs is gaining recognition, a comprehensive review focusing on the integration of passive EM during MI within the context of BCIs is still missing. Previous work has largely explored the cognitive correlates of MI-BCIs treating EM either as noise to be eliminated from EEG (Aggarwal and Chugh, 2019; Gu and Hua, 2021) or as a distinct control channel (e.g., using gaze for object selection) (Zhao et al., 2021). Consequently, research has frequently neglected the possibility of exploiting these concurrent EM signals as a passive window into the user’s cognitive state during MI (Lech et al., 2021). A primary explanation is that the temporal dynamics of EM during MI are not fully understood and it is still unclear which aspects of gaze data (e.g., dwell time, pupil dilation, blink rate) best indicate a user’s covert motor intentions (Fink et al., 2023; Tao et al., 2023). Additionally, though work has addressed the potential synergy between EM and motor commands, discrepancies exist regarding how EM should be integrated in MI-BCIs and whether these integration strategies generalize across users, training routines, and calibration methods (Ha et al., 2022; Meng et al., 2022).

Accordingly, the present work intends to clarify the role of EM during MI and constructs a synthesis of their integration into hybrid BCI paradigms. Our core proposition is that the relationship between EM and MI within a BCI context is functionally significant. We contend that monitoring EM during imagery opens a window into the underlying cognitive processes that reflect motor planning, attention allocation as well as maintenance, and perceived effort. We then propose that integrating this passively obtained EM data with EEG signals could lead to significant advancements in BCI technology. For instance, detecting pupil dilation indicative of high cognitive load could trigger an adaptive BCI to temporarily lower the task difficulty or adjust its decoding thresholds before the user becomes frustrated. Consequently, this paper integrates research that bridges cognitive understanding with practical BCI application. We move past treating a BCI as a pure control tool and instead frame it as an interaction framework in which the user’s cognitive state shapes how the system adapts. This perspective allows BCIs to infer richer aspects of user intention and adapt accordingly while simultaneously serving as a unique tool to investigate motor cognition itself in ecologically relevant settings.

## 2 Neurocognitive foundations linking motor imagery and eye movements

### 2.1 Neural basis of motor imagery

MI involves the internal rehearsal of a motor act without actual physical movement (Decety, 1996; Jeannerod, 1995; Kosslyn et al., 1995). Central to MI is the concept of functional equivalence, which postulates that imagined actions engage neural circuits similar to those used in the planning and execution of actual movements (Decety et al., 1994; Porro et al., 1996). Neuroimaging studies repeatedly show activation in a core network during MI that significantly overlaps with motor execution (Guillot et al., 2009; Hardwick et al., 2018; Monaco et al., 2020; Sharma and Baron, 2013). Prominent regions include secondary motor areas, such as the supplementary motor area (SMA) and the premotor cortex (PMC), as well as parietal regions like the superior and inferior parietal lobules (SPL/IPL).

While activation in the primary motor cortex (M1) is often weaker or actively suppressed compared to physical execution (Hétu et al., 2013), MI reliably engages frontoparietal and subcortical circuits crucial for spatial processing, working memory, and the inhibition of overt movement (Battaglia et al., 2006; Cengiz and Boran, 2016; Decety and Grèzes, 2006). Importantly for the present framework, these parietal and premotor circuits responsible for spatial planning and simulated sensory consequences in MI also constitute the anatomical substrates governing oculomotor control.

Also, the recruitment of shared neural substrates underscores intrinsic commonalities between MI and ME leading to significant functional consequences. Compelling evidence confirms that MI practice can lead to improvements in motor skills learning and performance yielding benefits comparable to physical practice (Frank et al., 2024; Sisti et al., 2022; Mizuguchi and Kanosue, 2017). The effectiveness of this approach stems from its capability to strengthen motor representations and stimulate neuroplastic changes, including modifications in cortical excitability and map reorganization, within the sensorimotor system through repeated rehearsal (Avanzino et al., 2015; Ruffino et al., 2017). As a result, MI’s characteristics make it a valuable tool in neurorehabilitation, offering a way for patients with motor impairments to engage motor networks and facilitate recovery (Nanbancha et al., 2023; Villa-Berges et al., 2023).

### 2.2 Oculomotor correlates of simulated action and gaze behavior

Neural activity offers an internal view of MI but the previously discussed shared anatomy implies that EM manifest a readily observable correlate of the underlying cognitive and motor simulation processes. The study of gaze behavior during MI builds upon a rich history of research connecting EM to perception, attention, and cognition (Findlay and Gilchrist, 2003; Liversedge and Findlay, 2000; Rayner, 1998) and is supported by two key theoretical frameworks. Foundational theories suggest that EM play a functional role not just in perceiving the world but also in constructing internal mental representations (Brandt and Stark, 1997; Kosslyn, 1994; Laeng and Teodorescu, 2002). Hebb’s (1968) scan path theory presents a prime example suggesting that visual imagery involves reinstating perceptual scan paths and reactivating the oculomotor commands stored alongside the visual memory. Analogously, theories of embodied cognition posit that cognitive processes like imagery are anchored in the body’s sensorimotor systems where actions, like EM, are viewed as being integral components of the cognitive architecture (Barsalou, 2008; Johansson et al., 2012; Spivey and Geng, 2001).

The close link between gaze and cognition is especially important for understanding MI, particularly in spatially guided tasks, as it closely relates to the principles of visuomotor coupling observed during overt ME. In executed goal-directed movements, particularly involving the hands, gaze typically arrives at key locations by predictively preceding the effector to gather visual information crucial for planning and control (Johansson et al., 2001; Pelz et al., 2001). The tight coupling between the eyes and the hand, essential for interacting with the environment (Land et al., 1999; Sailer et al., 2005), is partially preserved when actions are only imagined and it is rooted in the shared representations for both eye and limb control within the parietal and premotor circuits (Andersen and Buneo, 2002; Snyder et al., 1997). Additional behavioral evidence indicates that EM during MI often mirror the spatial characteristics and expected sequence of actual ME even when visual targets are absent or the eyes are closed (Heremans et al., 2008). These results directly support the functional equivalence hypothesis and indicate that MI activates both core motor representations as well as associated visuomotor coordination strategies.

However, the extent to which EM characteristics during MI replicate those during ME is dependent on task constraints. Even though overall congruency is maintained for predictable tasks targeting static objects (Heremans et al., 2008, 2009), a systematic breakdown is observed as task difficulty increases (Causer et al., 2013). For example, imagined movements constrained by Fitts’ Law may show appropriate overall time scaling, yet their fine-grained temporal gaze characteristics often do not match those of physical execution (McCormick et al., 2013). This effect becomes more pronounced in dynamic tasks requiring continuous prediction, such as intercepting a moving target, where the high cognitive load of maintaining the simulation and predicting outcomes without real-time sensory feedback can cause fundamental shifts in visuomotor control strategies (D'Aquino et al., 2023; Fooken et al., 2021).

Beyond mirroring the spatiotemporal organization of imagined actions, specific EM metrics also serve as real-time indicators of cognitive load and processing stages involved in MI. Changes in pupil size, often indicative of mental effort and arousal (Ebitz and Moore, 2019; van der Wel and van Steenbergen, 2018), consistently increase during MI tasks, likely reflecting the demands of simulating movement, maintaining the mental image, or estimating task parameters (Zekveld et al., 2018). Although fixation times may reflect processing time allocated to elements of the imagined scene or action (Vickers, 2007), saccadic parameters (e.g., latency, amplitude, and frequency) serve to distinguish active motor simulation from visual-perceptual processes (D'Aquino et al., 2022) while also revealing shifts in visuomotor control strategies necessitated by task dynamics (D'Aquino et al., 2023). Likewise, blink frequency, tied to attentional states and neurochemical activity, may offer another peripheral marker of the cognitive state during MI (Nakano et al., 2013; Oh et al., 2012).

In sum, MI is accompanied by externally observable oculomotor correlates. More precisely, these EM reveal that while the core principles of visuomotor coupling from ME are preserved, they are heavily modulated by the cognitive demands of the imagined task. Comprehending how these signals are generated, what information they reliably convey, and the challenges inherent in decoding them provides the necessary basis for exploring their synergistic application in BCIs.

## 3 Signals, paradigms, and integration strategies for decoding motor intentions

### 3.1 Fundamental signals for control and cognitive insight

At its core, a BCI operates by detecting and translating physiological signals reflecting user intention into actionable commands for external devices (Wolpaw and Wolpaw, 2012). For non-invasive BCIs, EEG stands as a cornerstone technology valued for its high temporal resolution and capability to measure cortical activation (Lotte et al., 2018; McFarland and Wolpaw, 2011). Yet, other signals that record oculomotor activity, such as metrics that are derived from EOG or eye-tracking (ET), are gaining recognition as valuable data channels in their own right (Dimigen and Ehinger, 2021; Karas et al., 2023).

The way EEG, EOG, and eye-tracking data are used defines a BCI’s operational mode which can be distinguished as active or passive (Zander and Kothe, 2011). Active BCIs depend on users deliberately altering their brain or physiological activity via a defined mental strategy (e.g., performing MI, directing visual attention, or making deliberate EM) to directly control the interface (Akram et al., 2015; Nicolas-Alonso and Gomez-Gil, 2012). The BCI decodes these voluntarily generated patterns into discrete commands (e.g., cursor movements) (Wolpaw et al., 2002). Instead, passive BCIs function by monitoring naturally occurring physiological fluctuations linked to the user’s cognitive state, attentional focus, or spatial context without requiring the user to exert deliberate control through those state changes (Alzahab et al., 2021; Kohlmorgen et al., 2007). Information acquired passively can then be used by the system to adapt the interface parameters, deliver feedback, or detect errors (Krol et al., 2018; Zander et al., 2010). Importantly, passive monitoring of a cognitive state can be implemented simultaneously with active BCI control to create a hybrid framework that can gain an accurate understanding of the user’s interaction context.

Building on this principle, the present paper proposes such a framework, with MI-EEG as the primary active control channel and passive EM as a continuous source of state information. Throughout the remainder of the paper, the term “passive” refers both to this BCI mode in Zander and Kothe’s (2011) sense and, more specifically, to EM signals that the user does not direct as commands; the latter use is formalized in Section 4.1. The full architecture, the active-passive distinction as it applies to EM, and worked examples are developed in Section 4. The remainder of the present section examines the limitations of MI-EEG decoding that motivate this integration.

### 3.2 Neural correlates and decoding challenges in MI-EEG

Despite MI being an intuitive strategy for BCI control, obtaining reliable performance based solely on EEG continues to be a major hurdle. This challenge stems from the nature of the neural patterns used for decoding: the sensorimotor rhythms (SMRs) recorded over cortical motor areas (Pfurtscheller and Neuper, 2001; Jeon et al., 2011; Müller-Putz et al., 2015). Executing or imagining movement often leads to a power decrease within the mu (8–12 Hz) and beta (18–26 Hz) frequency bands particularly over the contralateral hemisphere which is a phenomenon known as Event-Related Desynchronization (ERD) (McFarland and Wolpaw, 2005). Following MI, periods of motor inactivity or relaxation often exhibit a power increase in these bands termed Event-Related Synchronization (ERS) (Cassim et al., 2000). Through learning to consistently generate distinct ERD/ERS patterns by imagining movements of different limbs (e.g., left hand vs. right hand), users can generate control signals for the BCI (Blankertz et al., 2007; Wolpaw et al., 1991).

In practice however, translating these SMR modulations into dependable commands is a significant problem for several reasons (Lotte et al., 2018; Saha and Baumert, 2020). First, EEG signals suffer from a low signal-to-noise ratio (SNR) making them highly susceptible to contamination from both physiological and environmental artifacts (Goncharova et al., 2003; Jiang et al., 2019). Among these, EM-related artifacts from EOG are typically discarded regardless of their potential for hybrid BCI development. Second, SMR features exhibit high variability both between individuals (inter-subject) and within a single user over time (intra-subject). Across users, the precise frequency bands, topography, and magnitude of SMR modulation can vary significantly (Fazli et al., 2012; Ahn and Jun, 2015). For a single user, these neural signals can fluctuate due to variables like fatigue, shifts in attention, or changes in mental strategy, thereby requiring frequent recalibration or complex adaptive algorithms (Shenoy et al., 2006; Vidaurre et al., 2011; Arvaneh et al., 2013). Finally, a sizable percentage of users (estimated at 15–30%) cannot achieve proficient control of an MI-BCI even after extensive training - a phenomenon known as BCI illiteracy (Allison and Neuper, 2010; Vidaurre and Blankertz, 2010). Though the underlying neurophysiological factors are still under investigation, this issue likely derives from an inability to produce consistent and discernible SMR activity (Hammer et al., 2012).

Conventional signal processing methods have been developed to address the above challenges, yet they frequently fail to overcome the fundamental limitations of the EEG signal (Abiri et al., 2019). The combination of low SNR, high inter-subject variability, and inherent non-stationarity creates a performance ceiling that sophisticated algorithms alone, particularly outside of controlled laboratory settings, often cannot break. The resulting deficit in the primary control signal establishes a strong imperative to look beyond EEG-only solutions. Incorporating complementary data sources, such as passively monitored EM, therefore emerges not as an incremental improvement but as a necessary measure for bolstering the robustness and reliability of the BCI.

### 3.3 Integrating passive eye movements to enhance MI-BCI performance

The concept of augmenting MI-BCIs with passive EM is grounded in the availability of oculomotor metrics that function as online indicators of a user’s cognitive state. Unlike EEG, which requires complex decoding to reveal intent, EM grant immediate insight into the cognitive processes accompanying MI. Pupil dilation, a marker of cognitive load and mental effort tied to the noradrenergic system (Ebitz and Moore, 2019; van der Wel and van Steenbergen, 2018), permits a BCI to distinguish between task engagement and disengagement even in the absence of clear EEG patterns. This is relevant for differentiating a user who is struggling to generate an MI signal from one who is not attending to the task (Voudouris et al., 2023; Zekveld et al., 2018).

Furthermore, the analysis of fixations and saccades provides a spatial reference for the neural data. Since gaze characteristics often reflect the spatial aspects of imagined actions (de'Sperati, 2003; Land et al., 1999), fixation locations can furnish probabilistic evidence for a user’s intended spatial goal. This empowers the decoder to verify decoded EEG intentions or track spatial attention before a motor command is triggered (Johansson et al., 2001). Similarly, blink patterns and saccadic dynamics contribute supplementary data on vigilance and fatigue (Eckstein et al., 2017) thus ensuring the decoder adapts to the user’s waning attention over time. The architectural advantages this affords are formalized in Section 4.

From a technical perspective, realizing this hybridity relies on a framework for fusing multimodal data streams (Hong and Khan, 2017; Pfurtscheller et al., 2010). Signal fusion may happen at various stages combining feature-level fusion, which concatenates EEG and EM vectors to capture non-linear inter-modal dependencies, and decision-level fusion, which combines independent classifier outputs for greater modularity (Cheng et al., 2020). Although the former method might better capture inter-modal dependencies, the latter offers greater modularity and robustness if one modality fails (Fazli et al., 2009). Increasingly, deep learning architectures are being employed to automatically learn the non-linear latent representations that link neural oscillations with oculomotor dynamics (Roy et al., 2019; Rudroff, 2025; Zhang et al., 2020).

The practical implementation of passive EM monitoring presents obstacles that extend beyond the technical requirement for precise temporal synchronization (Jamal et al., 2023). A major challenge lies in preserving the ecological validity of the user’s behavior. The system faces the difficult task of using eye data without inadvertently incentivizing the user to manipulate their gaze for explicit control. To function as a truly passive support system the interface must successfully extract intention from natural oculomotor behavior without imposing additional cognitive load or forcing the user to deviate from their intuitive visual strategy (Fairclough, 2009). Resolving these theoretical and technical constraints requires examining how multimodal fusion is operationalized in real-world scenarios. Therefore, the subsequent analysis of existing hybrid implementations serves to distinguish between established active control paradigms and the emerging use of passive cognitive monitoring.

## 4 Proposed framework for passive EM integration in MI-BCIs

### 4.1 Distinction between passive and active EM in MI-BCIs

A persistent source of ambiguity in hybrid BCI research concerns what it means to label EM as passive or active. Even when a user is not deliberately operating an interface controlled by gaze, oculomotor behavior unfolds in causal response to the task at hand: fixations support motor planning, saccades sample locations relevant to the task, and pupil dilation tracks the cognitive demand of the moment. In this sense, EM during MI could be considered active, because they are causally linked to a voluntarily generated mental event. Yet, the same EM also fall outside the conventional definition of an active BCI signal, because the user does not modulate them with the explicit intent of producing a command. Clarifying which sense of active or passive applies is therefore necessary before the framework can be operationalized.

Two alternative interpretations of the active–passive distinction deserve consideration before adopting a working definition. The first equates passive with involuntary, in which case the label would apply only to reflexive oculomotor events such as the pupillary light reflex while task-related fixations and pursuits would be classified as active. We find this interpretation too restrictive, as most of the EM that carry useful information during MI emerge in response to task demands and are not involuntary in any strict sense. The second interpretation defines passive in anatomical terms, on the grounds that EM lie outside the primary motor circuit engaged during the imagined act. This captures part of what we mean by passive EM but remains problematic, since the parietal and premotor circuits governing oculomotor control overlap substantially with those engaged during MI of manual actions (Andersen and Buneo, 2002; Snyder et al., 1997), as discussed in Section 2. EM during MI are generated by the same broader visuomotor system that supports the imagined movement, and a clean anatomical separation between the two is not available.

We therefore adopt a third, intent-based interpretation where EM qualify as passive when the user does not direct them with the purpose of conveying a command to the BCI. The system extracts information from oculomotor behavior that the user produces as part of engaging with the task, rather than from gaze that the user has been instructed to deploy as a control input. By this definition, a fixation that supports the user’s own motor planning is a passive signal for the BCI, whereas the same fixation deliberately held on a target to trigger a selection would be active. This functional reading aligns with the broader distinction between active and passive BCI modes introduced earlier (see Section 3.1; Zander and Kothe, 2011) and clarifies why concurrent EM during MI represent an underexploited resource rather than a competing control channel. It also supports the architectural separation that the next subsection makes explicit, in which passive EM contextualize the active EEG command stream without requiring the user to deploy gaze for a second control purpose.

### 4.2 Proposed framework

**Figure 1:**
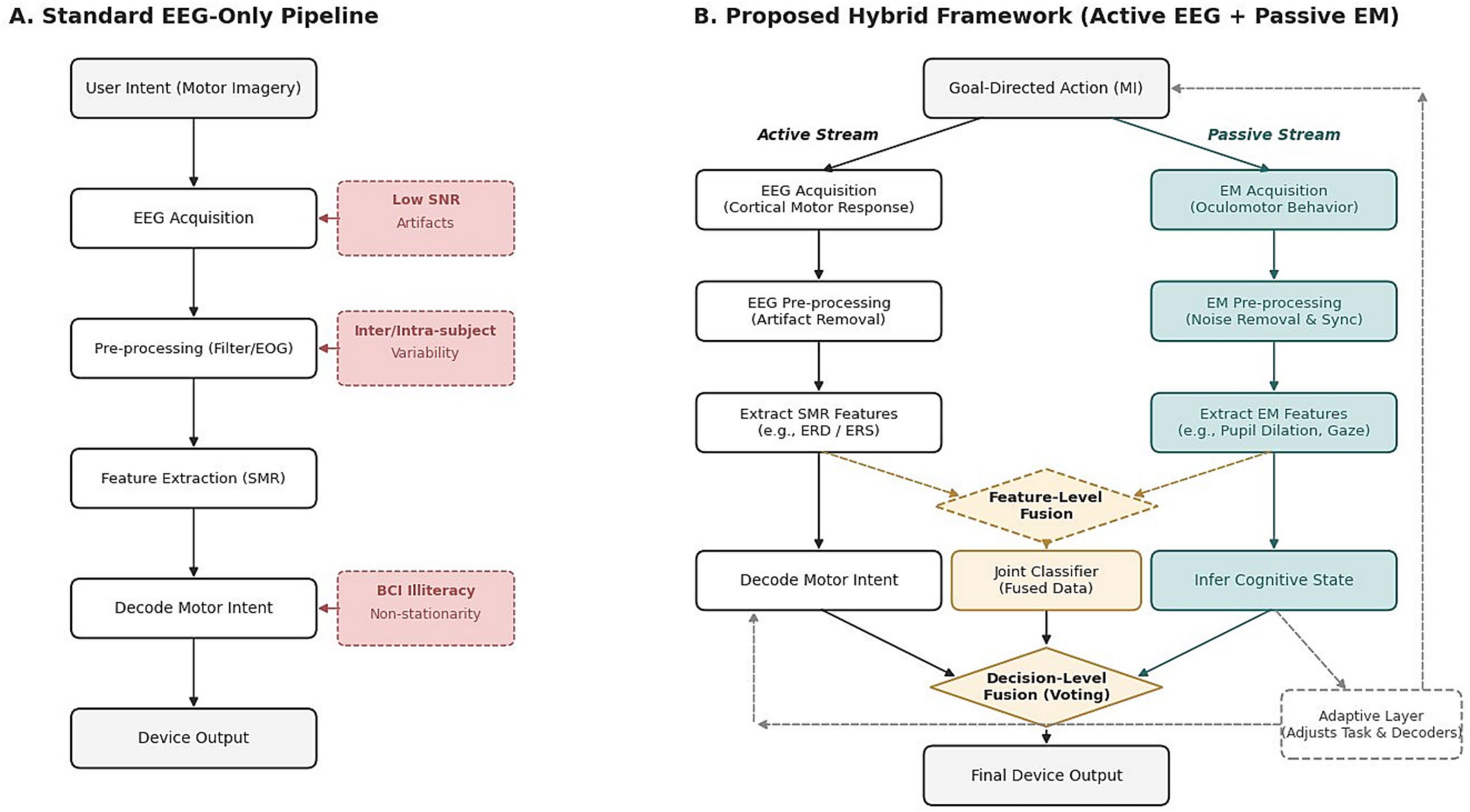
Architectural comparison between standard and hybrid BCI pipelines. **(A)** The standard EEG-only pipeline operates as a single stream where inherent limitations (e.g., low signal-to-noise ratio, artifacts, and high variability) propagate downstream, frequently leading to unreliable device output. **(B)** The proposed hybrid framework integrates an active neural stream (EEG) with a parallel passive oculomotor stream (EM). The framework supports both feature-level fusion (dashed gold arrows) and decision-level fusion (solid black arrows). Furthermore, the passive stream continuously informs an adaptive feedback loop (dashed gray arrows), adjusting decoder thresholds and task properties in real time to compensate for fluctuations in the user’s cognitive state.

Building on the distinction between passive and active EM, we propose a hybrid architecture in which MI-EEG functions as the primary active channel for volitional control while passive EM provide a continuous secondary stream of cognitive state information. The framework comprises four functional stages illustrated in Figure 1.

In the first stage, the user performs MI of a goal-directed action while the system simultaneously acquires both EEG and EM signals. In the second stage, EEG features are used to decode motor intent, typically through ERD/ERS modulations over sensorimotor areas (McFarland and Wolpaw, 2005; Pfurtscheller and Neuper, 2001), while passive oculomotor metrics (e.g., pupil diameter, fixation patterns, smooth pursuit characteristics, and saccadic dynamics) are processed in parallel to infer the user’s cognitive state. In the third stage, the two streams are combined through multimodal fusion, which can occur at the feature level or at the decision level (Cheng et al., 2020; Fazli et al., 2009; Hong and Khan, 2017). In the fourth stage, the fused output drives the device while continuously informing an adaptive layer that adjusts decoder parameters and task properties in response to fluctuations in the inferred cognitive state. Although hybrid systems may also incorporate EM as an active control channel, such as gaze for target selection or EOG for directional commands (Ha et al., 2022; Putze et al., 2019; Zhang et al., 2019), our framework deliberately reserves the active role for MI-EEG to preserve the user’s natural visuomotor behavior and avoid imposing the demands of dual control.

Within this architecture, each oculomotor metric serves a defined role grounded in the evidence reviewed in Section 3.3. Pupil diameter indexes cognitive load and task engagement (Ebitz and Moore, 2019; Voudouris et al., 2023). Fixations carry spatial information that can verify or constrain decoded intent (Ge et al., 2023; Johansson et al., 2001). Smooth pursuit reflects the visuomotor strategy adopted during covert motor processes (D'Aquino et al., 2022, 2023). Saccadic dynamics and blink patterns index vigilance and fatigue (Eckstein et al., 2017). In parallel, even if the framework is articulated here around EEG and EM, the same architecture readily accommodates other physiological correlates of cognitive state, such as heart rate variability, as additional passive channels (Aricò et al., 2018). It must be noted that pupillary and cardiovascular markers are not interchangeable. Pupil diameter responds on the order of hundreds of milliseconds to changes in cognitive load and noradrenergic activity, making it well suited to track moment-to-moment fluctuations in effort during MI. By contrast, heart rate variability operates over longer time windows and is more sensitive to sustained shifts in autonomic state, such as accumulated fatigue or arousal across a session. In the proposed framework, the two are complementary as pupillometry feeds the ongoing adaptation of decoder parameters, while heart rate variability could inform more long-term decisions such as scheduling rest breaks or adjusting session length. The same logic extends to other autonomic markers (e.g., galvanic skin response) whose temporal characteristics determine their best use within the architecture.

The integration of passive EM features confers several direct advantages for BCI performance. One benefit is enhanced accuracy and reliability, where EM features can help disambiguate noisy or weak EEG signals by supplying complementary contextual information and by extending crucial support for users struggling with BCI illiteracy or during periods of high cognitive load (Allison and Neuper, 2010). Furthermore, it improves overall system robustness. Since EM signals typically possess a higher signal-to-noise ratio than EEG and are less susceptible to contamination from artifacts like EMG, their inclusion strengthens the overall architecture (Kilicarslan and Contreras-Vidal, 2019). This robustness extends to techniques capable of repurposing oculomotor signals (e.g., EOG), traditionally discarded as noise, to operate as an informative data channel (Karas et al., 2023; Tao et al., 2023). Also, passively acquired state information allows the BCI to infer if a user is fatigued, struggling, or inattentive, and to adapt task parameters or feedback accordingly to ensure a less frustrating experience (Aricò et al., 2016; Gerjets et al., 2014). Finally, integrating EM improves intention verification. Gaze cues associated with planning can also be used to cross-check the intention decoded from EEG thereby mitigating the Midas Touch problem where unintended neural fluctuations trigger unwanted commands (Ge et al., 2023; Shishkin et al., 2016).

### 4.3 From conceptual paradigm to clinical practice

To make the framework concrete, we now consider two scenarios that illustrate how passive EM can support MI-BCI operation under different conditions. The first is a controlled experimental task and the second is a representative clinical application.

**Figure 2:**
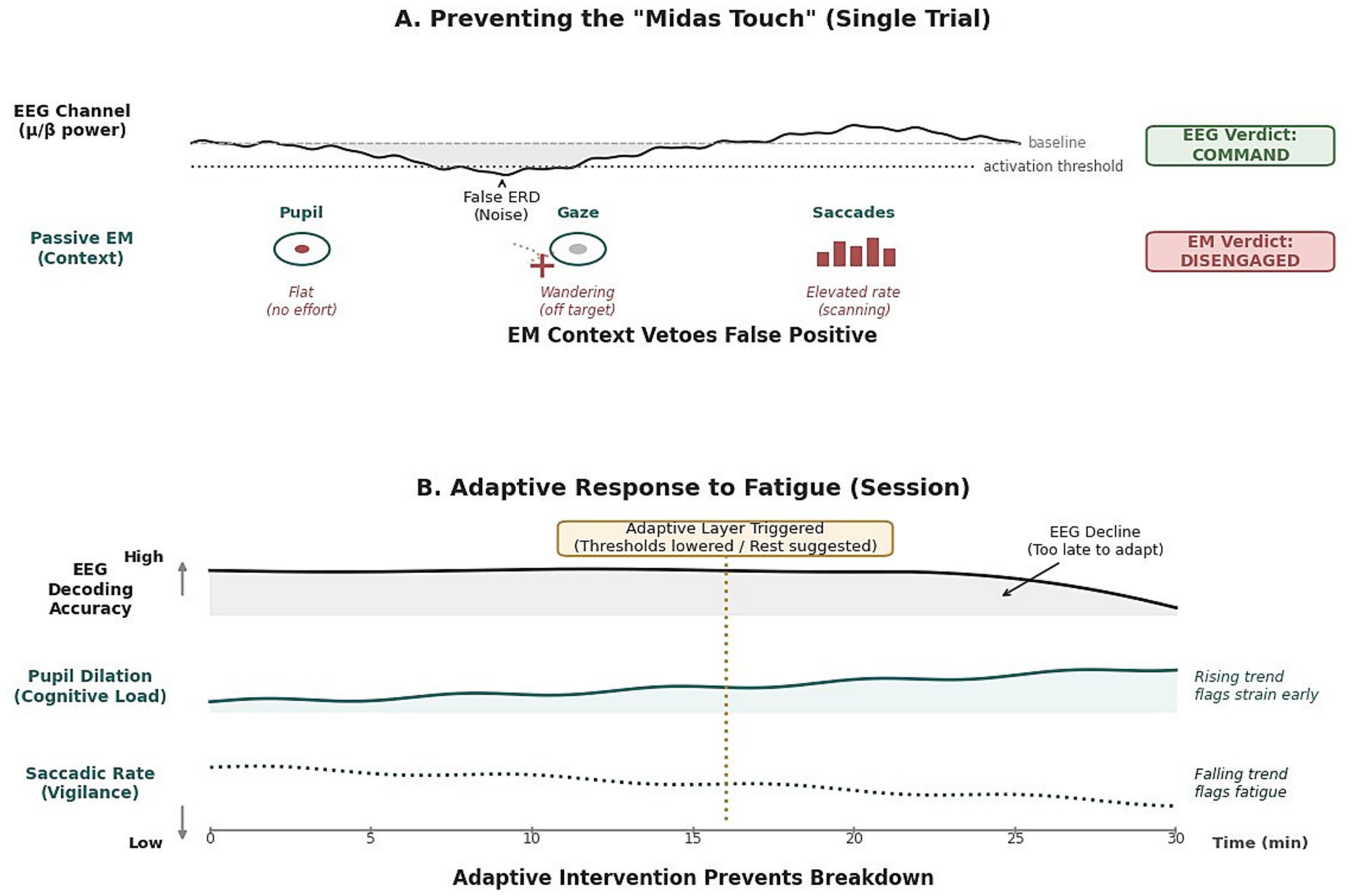
Two scenarios demonstrating the operational benefits of integrating passive EM. **(A)** Over a short timescale (single trial), the system prevents the Midas Touch. Even if EEG noise falsely crosses the activation threshold, concurrent EM metrics (in red) contradict the neural signal, allowing the decision-level fusion to veto the false positive. **(B)** Over a long timescale (continuous session), the system exhibits an adaptive response to fatigue. In this instance, EM metrics act as early warning signals of cognitive fatigue. This tracking triggers the adaptive layer to adjust task parameters or decoder thresholds before the user’s primary EEG performance degrades.

In the experimental scenario (Figure 2A), the user performs MI of an interception, mentally simulating the act of catching a moving target. In this instance, EEG decodes the user’s motor intent through ERD/ERS modulations over sensorimotor areas. In parallel, the system tracks oculomotor behavior. Smooth pursuit characteristics indicate whether the user is genuinely engaged in motor simulation, since covert motor processes during interception modulate gaze in ways that differ from passive observation of the same target (D'Aquino et al., 2022; Spering and Montagnini, 2011). Fixations and saccadic dynamics provide complementary information on spatial attention and the interceptive strategy adopted by the user (D'Aquino et al., 2023; Fooken et al., 2021). Pupil diameter signals the cognitive effort invested in the task. The fusion stage combines these streams and the system can confirm whether the decoded EEG intent reflects an active simulation or a momentary lapse in engagement before triggering any output.

In the clinical scenario (Figure 2B), a stroke survivor undergoes upper-limb rehabilitation supported by an assistive device, such as a robotic arm or an exoskeleton. The patient performs MI of a grasping or reaching action, and the BCI must decode this intent reliably enough to initiate assistance from the device (Cervera et al., 2018; Colucci et al., 2022; Soekadar et al., 2016; Wang et al., 2023). Therapy sessions, however, frequently extend beyond the user’s optimal attentional window, and EEG signals deteriorate as fatigue and disengagement accumulate (Belkhiria et al., 2022). Passive EM provide the system with a window onto these state changes. Pupillary responses can flag rising cognitive load before EEG performance degrades (Ebitz and Moore, 2019), saccadic dynamics and blink patterns can indicate fatigue (Eckstein et al., 2017), and fixation behavior can confirm spatial attention to the target before assistance is triggered. The adaptive layer of the framework can then simplify the task, adjust the decoder threshold, or suggest a rest break (Krol et al., 2018; Zander and Kothe, 2011), supporting longer and more productive sessions in less supervised settings such as home-based rehabilitation (Simon et al., 2021).

Taken together, these two scenarios are not exhaustive, but they show that the framework operates consistently across paradigms. Whether the goal is to disambiguate covert motor processes in an experimental task or to maintain reliable assistive control in a therapeutic context, passive EM provide the system with information that EEG alone cannot supply. The next section examines how existing hybrid implementations have begun to operationalize this principle.

## 5 Hybrid EEG-EM brain-computer interfaces: implementations and insights

### 5.1 Hybrid systems: integrating EEG and eye movements

The combination of EEG and EM has evolved along two divergent operational lines involving active control and passive monitoring. Most existing literature focuses on active control where EM function as an explicit input channel to augment EEG. In these implementations, integrating EOG or ET with EEG paradigms like SSVEP or MI accelerates interaction speed and expands the command set available to the user (Ha et al., 2022; Kim et al., 2015; Zhang et al., 2023). For example, systems combining EEG for primary intent with EOG for directional commands or blink detection allow for complex interactions, such as navigating virtual environments or manipulating robotic prostheses, far exceeding the capabilities of single-modality systems (Antoniou et al., 2021; Zhang et al., 2019).

In contrast, a separate line of inquiry aligns with our proposed framework by using passively monitored EM features as a continuous window into the user’s cognitive state. Rather than treating gaze as a deliberate command, these studies extract physiological markers to contextualize the neural data. Passively measured pupil diameter, for instance, markedly enhances a BCI’s ability to distinguish MI from rest and reliably reflects task complexity even during active control (Guo et al., 2023; Rozado et al., 2015). Similarly, incorporating passive fixation duration and saccadic metrics improves the classification of attentional states and visual targets, providing a stabilizing secondary signal independent of cortical motor rhythms (Ge et al., 2023; Vortmann et al., 2022).

Finally, recent research has begun to introduce advanced interaction designs that synchronize gaze-based selection with neural commands to tackle real-world usability. Dillen et al. (2024) created a shared control system in Augmented Reality (AR) where users selected objects with gaze and subsequently used MI-EEG commands to trigger robot actions. This design led to high task success rates for complex manipulation showing that well-designed hybrid interactions can compensate for poor underlying MI decoding. Venot et al. (2024) concentrated on the temporal dynamics of such interactions investigating how the timing of an MI command relative to a gaze-guided robot movement affected performance. They found that performing MI after the robot’s initial movement produced superior accuracy improvements and stronger neural responses. Taken together, these results show that EM serve a dual role in hybrid BCIs, both as passive markers of cognitive state that stabilize and contextualize the interaction, and as active inputs that expand the command set (see Table 1).

**Table 1:** Summary of the key studies discussed in Section 5.1 that integrate EEG with EM signals.

Study reference (Author, Year)	EEG paradigm	Modalities combined	EM Role	Specific EM metrics/signals used	Task	Key outcome/relevance
Kim et al. (2015)	Concentration State	EEG + ET	Active control (cursor pointing)	Gaze position	Pointing & selection task	Feasibility of hybrid low-cost system, BCI selection comparable to keyboard
Rozado et al. (2015)	MI	EEG + ET	Passive monitoring	Pupil diameter	MI vs. rest classification	Improved MI accuracy, easier BCI utilization
Soekadar et al. (2016)	Sensorimotor rhythms / Intention detection	EEG + EOG	Active control (EEG/EOG for hand exoskeleton)	EOG signals	Control of hand exoskeleton for daily activities	Restored independent daily living after quadriplegia
Zhang et al. (2019)	MI	EEG + EOG + EMG	Active control (directional Cmds, blink switch)	Directional EOG (look L/R), voluntary double blinks	Soft robot hand control	Expanded command set
Cheng et al. (2020)	MI	EEG + ET	Passive monitoring (EM data fusion for MI classification)	Eye movement features (fixation, saccade, pupil)	MI classification	Improved MI classification accuracy
Antoniou et al. (2021)	General EEG patterns	EEG + EOG	Active control	EOG signal patterns (L/R/U/D movements, Open/Closed states)	EM classification	EM commands decodable from scalp
Ha et al. (2022)	SSVEP	EEG + EOG	Active control (horizontal selection)	Directional EOG features (L/M/R classification)	VR navigation	Improved accuracy & ITR
Vortmann et al. (2022)	Attention state	EEG + ET	Passive monitoring	Saccade velocity, fixation count, pupil diameter	Internal vs. external attention classification	Late/Middle fusion best for EEG + passive ET state classification
Ge et al. (2023)	ERPs	EEG + ET	Passive monitoring	Fixation duration	Visual Search Target Identification	Improved ERP-based intention detection
Guo et al. (2023)	SSVEP/EOG Control	EEG + EOG + ET	Active control (EOG) & passive monitoring (ET)	Directional EOG (control), pupil diameter (passive monitor)	Cursor control task/robot arm control	Passive pupil diameter tracked cognitive load during active control
Lin et al. (2023)	Grasp intention state	EEG + ET	Passive monitoring	Pupil mean diameter, pupil amplitude change, saccades	Grasp intention classification	EEG-ET coupling during grasp intention
Zhang et al. (2023)	SSVEP	EEG + EOG	Active control (region selection)	Directional EOG features (region classification)	Spelling task	Improved accuracy & ITR
Dillen et al. (2024)	MI	EEG + ET	Gaze selection (object)	Gaze position	Robotic arm pick-and-place in AR	High task success rate
Venot et al. (2024)	MI	EEG + ET	Gaze selection (target)	Gaze position	Robotic arm reach-and-grasp	Improved BCI accuracy, stronger & more localized motor-related brain activity
Yi et al. (2024)	P300	EEG + ET	Passive monitoring & gaze selection	Pupil diameter, fixation/saccade metrics, gaze position	Communication task (DOC patients)	Improved classification accuracy

Columns detail the main BCI paradigm, combined signal modalities, EM metrics, the experimental task performed by participants, and the primary outcome of the study. DOC, Disorders of Consciousness; ITR, Information Transfer Rate.

### 5.2 Advanced signal processing and machine learning for fusing passive eye movements with MI-EEG

Hybrid EEG-EM systems share a common processing pipeline that proceeds through preprocessing of each modality, feature extraction, multimodal fusion, and classification, with temporal alignment of the two streams as a precondition for any cross-modal operation (Jamal et al., 2023). On the EEG side, raw signals are typically band-pass filtered to the frequency bands relevant for sensorimotor activity and submitted to artifact handling techniques like Independent Component Analysis (ICA), Artifact Subspace Reconstruction, or regression-based correction (Jiang et al., 2019; Kilicarslan and Contreras-Vidal, 2019). EOG-related activity occupies a unique position in this pipeline. In conventional unimodal decoding, ICA or regression are deployed exclusively to discard ocular artifacts from cortical data. However, hybrid frameworks use these same spatial filtering techniques to isolate the ocular components and redirect them into an independent feature stream. By systematically separating the sources, the ocular signals that typically contaminate the EEG are preserved and processed in parallel to extract task-relevant kinematics such as blink frequency or saccadic amplitude (Karas et al., 2023; Tao et al., 2023). On the EM side, preprocessing routines detect blinks, segment the gaze trace into fixations and saccades, correct for drift, and compute pupil size baselines (Holmqvist et al., 2011). Once cleaned, both streams undergo feature extraction adapted to their respective dynamics, followed by fusion at one of several possible levels and classification through traditional or deep learning models. The architecture introduced in Section 4.2 maps onto this technical pipeline at the level of the first three stages, while the adaptive layer draws on features generated throughout.

Beyond simple data aggregation, the pipeline aims to identify synergistic relationships where subtle EM lend context for the neural activity underlying planning and execution (Arpaia et al., 2022; Hong and Khan, 2017; Lin et al., 2023). Hence, employing advanced feature extraction is useful to isolate relevant features from both streams. Common Spatial Patterns (CSP) and its variants remain the standard for discriminating MI states in EEG (Blankertz et al., 2007). In contrast, recent studies investigate obtaining richer neural data via spatio-spectral temporal features or source imaging techniques (Ang and Guan, 2017; Lotte et al., 2018). Parallel approaches for EM encompass detailed morphological analysis of EOG signals (Tao et al., 2023), the modeling of dynamic pupillary responses linked to cognitive effort (Fink et al., 2023; Rozado et al., 2015), and the analysis of fine-grained saccade and fixation kinematics (Rayner, 2009). Besides, innovative techniques facilitate the classification of task-related EM directly from EEG and EOG channels (Antoniou et al., 2021).

Once features are extracted, selecting an appropriate fusion strategy becomes the critical determinant of overall performance. Three broad approaches dominate the literature. Feature-level (early) fusion concatenates the feature vectors from each modality before classification, allowing the model to learn cross-modal correlations within a single representation (Cheng et al., 2020). Intermediate (middle) fusion processes each modality through dedicated layers before merging at an intermediate depth, after which shared layers complete the classification; recent variants implement this through cross-modal attention to dynamically weight modality contributions (Vortmann et al., 2022; Yi et al., 2024). Decision-level (late) fusion trains independent classifiers per modality and combines their outputs through fixed decision rules, evidence-theoretic schemes, or learned aggregation (Hong and Khan, 2017). The three strategies differ markedly in classification accuracy, in their tolerance to degradation of a single modality, and in computational cost. Table 2 provides a comparative summary across these dimensions, drawing on MI and adjacent EEG-EM paradigms because the methodology transfers across paradigms while MI-specific fusion evidence remains sparse; direct numerical comparison across studies is constrained by differences in paradigm, baseline, and reporting conventions.

**Table 2:** Comparative overview of multimodal fusion strategies for hybrid EEG-EM systems with relevance to MI-BCIs.

Fusion strategy	Mechanism	Representative studies	Reported accuracy	Robustness	Latency/computational cost
Feature-level (early)	Concatenation of feature vectors from each modality before a single classifier	Cheng et al. (2020) (MI); Vortmann et al. (2022) (attention); Lin et al. (2023) (grasp intention)	Cheng et al. (2020): feature-level fusion exceeded both unimodal baselines for MI. Vortmann et al. (2022): 60.8% (image features) and 57.2% (time-series) vs. 63.5% EEG-only, indicating sensitivity to feature representation	Sensitive to single-modality degradation; requires aligned, structurally compatible features	Single classifier pass at inference; lowest computational cost among multimodal approaches
Intermediate (middle)	Modality-specific layers process each stream independently before merging at intermediate depth; shared layers complete the classification	Vortmann et al. (2022) (attention); Yi et al. (2024) (P300, DOC)	Vortmann et al. (2022): 61.7%, intermediate between early-fusion variants and late fusion, not significantly different from either. Yi et al., 2024: multimodal-attention modules improved classification accuracy in DOC communication	Tolerates partial degradation of one stream because modality-specific branches preserve unimodal information; gains depend on architecture design	Higher than feature-level; sensitive to network depth; deep architectures may need GPU for real-time use
Decision-level (late)	Independent classifiers per modality; outputs combined through fixed rules, evidence-theoretic schemes, or learned aggregation	Cheng et al. (2020) (MI); Vortmann et al. (2022) (attention)	Cheng et al. (2020): late > feature-level for MI; combining EM with 50% of EEG channels reduced MI accuracy by ~1%. Vortmann et al. (2022): 64.2%, the highest accuracy and the only fusion strategy to significantly exceed both unimodal baselines	Highest tolerance to single-modality degradation; modular per-modality classifiers facilitate updates and recalibration	Two classifier passes plus aggregation; modular structure supports parallelization

Rows organize the literature by fusion level rather than by individual study to clarify trade-offs across approaches. Studies are drawn from MI and adjacent EEG-EM paradigms (attention, P300), as MI-specific fusion evidence is limited and methodological insights transfer across paradigms, consistent with the framing of Section 5.1. Reported accuracies are taken from the original studies; direct numerical comparison is constrained by differences in paradigm, baseline, window length, and reporting conventions, and the values should be read as within-study evidence rather than across-study benchmarks. Robustness and latency are characterized qualitatively because few studies report them quantitatively in comparable form. DOC, Disorders of Consciousness; EM, Eye Movements; MI, Motor Imagery.

To implement these fusion strategies, advanced classifiers are notably beneficial. For example, Shrinkage Linear Discriminant Analysis (SKLDA) has proven effective in hybrid systems combining EEG and eye tracking (Ge et al., 2023). However, deep learning architectures are becoming increasingly prominent due to their ability to automatically learn hierarchical features from high-dimensional data. Convolutional Neural Networks (CNNs) are frequently employed to capture spatial patterns while Recurrent Neural Networks (RNNs/LSTMs) efficiently model temporal dependencies within multimodal datasets (Schirrmeister et al., 2017; Roy et al., 2019; Craik et al., 2019; Lawhern et al., 2018). More recent architectures, such as the multimodal attention modules used by Yi et al. (2024), allow dynamic weighting of EEG and ET features offering the capability to adapt to changing signal quality and task demands.

Another step in the management of inherent non-stationarity and variability present in EEG signals calls for adaptive algorithms (Arvaneh et al., 2013; Vidaurre et al., 2011). A major advantage of integrating passive EM is that features reflecting real-time attention, fatigue, or cognitive load can act as direct inputs to these adaptive mechanisms (Guo et al., 2023; Lin et al., 2023; Rozado et al., 2015; Vortmann et al., 2022). Accordingly, the BCI can dynamically modify decoding strategies, interface parameters, or feedback contingent on an inferred user state. The possibility to do so facilitates the creation of user adaptive interfaces that remain responsive to cognitive fluctuations during extended MI tasks (Gerjets et al., 2014; Borghini et al., 2014; Krol et al., 2018).

### 5.3 Key applications in neurorehabilitation and assistive technologies

The translation of hybrid BCI technology from laboratory settings into viable clinical tools is a central area of development. In the domain of neurorehabilitation, patients suffering from stroke, spinal cord injuries, amyotrophic lateral sclerosis, or similar neuromuscular conditions may gain from assistive technology that enhances communication and mobility (Daly et al., 2013; Daly and Wolpaw, 2008; Müller-Putz et al., 2016; Saibene et al., 2023). Conventional MI-BCIs are able to engage the sensorimotor system but their clinical application is frequently hindered by the performance limitations discussed previously. Incorporating passive EM creates a compensatory mechanism that mitigates these targeted clinical deficits by providing a secondary input channel to stabilize control and monitor patient state.

A key contribution of augmenting MI-EEG with passive EM is the strengthening of control during challenging rehabilitation tasks. Therapy sessions for stroke or spinal cord injury patients often result in reduced attention and increased fatigue over time inevitably degrading BCI performance. Passive EM monitoring delivers a solution by detecting such indicators of cognitive states through metrics like blink frequency or saccade velocity (Belkhiria et al., 2022). Rather than the classifier misinterpreting commands due to state fluctuations (Arvaneh et al., 2011), an adaptive interface could simplify the task, increase decoder sensitivity, and eventually suggest a rest break (Zander and Kothe, 2011; Krol et al., 2018). State-aware responsiveness mitigates frustration allowing individuals to remain motivated and engaged for longer periods as well as eases the steep learning curve of BCI skills - indispensable for enabling independent use in less supervised environments like home-based rehabilitation (Simon et al., 2021).

Beyond immediate control, passive EM integration opens new pathways for objective monitoring and personalization of therapy. Inducing beneficial neuroplasticity through targeted MI practice is fundamental to recovery (Nanbancha et al., 2023; Ruffino et al., 2017). However, assessing the quality of a patient’s mental engagement has traditionally been subjective. Passive EM yield objective non-invasive biomarkers to overcome the reliance on self-reporting. For example, pupillometry provides a real-time quantification of the cognitive load associated with specific MI tasks (Voudouris et al., 2023). Translating this into practice, Guo et al. (2023) deployed a real-world hybrid system for robotic arm control in a VR environment. Their system passively monitored pupil diameter to track the user’s cognitive load continuously instead of using gaze solely for active targeting. This implementation allowed the system to objectively quantify mental effort without interrupting the primary BCI control channel, proving that passive pupillometry can effectively run in parallel with active neural decoding to contextualize the user’s performance. Such insight enables clinicians to adapt the intensity of therapy based on objective effort indicators, especially when combined with immersive technologies including VR and AR (Alimardani and Hiraki, 2020; Said et al., 2022).

In terms of usability, hybrid BCIs using EM can facilitate safer and more reliable control of assistive devices. Ensuring intentional and accurate commands is a core necessity for operating systems that include robot arms (Venot et al., 2024), exoskeletons (Colucci et al., 2022), wheelchairs (Li et al., 2013; Wen et al., 2021), and neuroprostheses (Wang et al., 2023). An early demonstration of clinical viability comes from Soekadar et al. (2016), whose hybrid EEG/EOG hand exoskeleton restored fully independent daily living activities for patients with quadriplegia. In these high-risk environments, passive EM cues related to visuomotor planning can act as a verification signal to cross-check the output of the MI decoder before execution. One example is in the context of wheelchair navigation where hybrid systems have successfully employed passive fixation data to verify intended commands, significantly reducing the likelihood of errors and neutralizing the Midas Touch effect in complex naturalistic tasks (Gegenfurtner, 2016; Li et al., 2013; Shishkin et al., 2016). Ultimately, integrating passive EM advances the field toward the seamless interaction seen in multi-input systems (Zhang et al., 2019) that are more robust, adaptive, and clinically meaningful (Saibene et al., 2023; Vortmann et al., 2022).

## 6 Challenges, methodological implications, and future directions

### 6.1 Integrating passive eye movements with MI-BCIs

The neurocognitive synergy between the eyes and motor systems is clear; however, translating this relationship into practical applications presents engineering barriers. Among these is the nuance of feature selection. On one hand, researchers have established that metrics including pupil diameter, fixation durations, and saccadic parameters correlate with cognitive states. On the other hand, ascertaining precisely which features yield dependable insights about the covert processes of MI stands as an open question. Complicating matters further is the lack of specificity unique to these metrics as they are not exclusively tied to MI. For instance, increased pupil dilation signals effort, but fails to address whether that effort is directed toward the motor task or arises from an unrelated distraction. Even environmental factors might play a role. Distinguishing whether a pupil dilation stems from mental effort or simply the pupillary light reflex triggered by a shadow mandates robust signal separation techniques. Moreover, the precise nature of the motor task dictates the utility of these signals. Since the coupling between gaze and cognition is most robust during spatially guided actions, researchers must accept that passive EM features may prove less informative for purely kinesthetic forms of imagery. Without careful filtering this ambiguity introduces a substantial possibility of adding noise or incorporating features that possess only a spurious correlation with MI performance.

Equally significant is the computational barrier posed by cross-modal fusion. Merging passive EM signals with active intentionally modulated EEG during MI is not just a data aggregation task but demands advanced processing methods. Standard approaches often fail here because EEG and EM operate on vastly different timescales and exhibit distinct signal characteristics. Basic implementations of these strategies, such as naive concatenation or fixed-rule decision merging without modality coupling considerations, frequently miss the subtle interplay between the user’s underlying cognitive state and their capacity to generate discriminable MI patterns. High cognitive load could simultaneously increase pupil dilation and suppress specific EEG rhythms, potentially leading to misinterpretation by standard classification methods. Hence, the success of this hybrid model largely depends on developing algorithms capable of modeling intricate cross-modal dependencies that vary across time.

Apart from data processing, temporal dynamics and synchronization introduce a layer of ambiguity regarding the causal sequence of neural and behavioral events. While accurate alignment of high-resolution EM data with EEG is a technical prerequisite, the interpretation of their temporal relationship requires deciphering which signal drives the other. Researchers face the nontrivial decision of discerning whether a change in EM functions as a predictive indicator of a preceding cognitive state or acts as a reflection of effort with the purpose of pinpointing the precise moment a BCI should intervene to assist the user. Another important consideration is balancing the algorithmic interpretation of the state with the risk of control interference. Specifically, the goal is to use passive EM for adaptation but an undesirable interpretation by the system could clash with the user’s deliberate MI command with the threat of undermining the user’s sense of agency. If the algorithm interrupts a user’s learning process driven by gaze data, it risks becoming intrusive. Therefore, a careful balance is required to leverage state information without overriding the user’s primary intent. Finally, the feasibility of a universal monitoring model can be limited by the fact that oculomotor patterns during MI exhibit significant variation both between users and across different experimental contexts. Overcoming this issue requires a shift toward more personalized calibration strategies capable of adapting to each user.

### 6.2 Methodological implications for BCI research and development

Adopting the perspective that passive EM offers a window into cognitive state necessitates an improvement of standard protocols towards multimodal experimental paradigms designed to simultaneously record actively generated neural signals and passively evolving indicators of attention. Current frameworks often focus only on the success rate of the motor command and neglect the physiological context in which that command was generated. To map how some EM change in response to task difficulty, task designs must explicitly produce relevant variations in both MI control and cognitive load.

To this end, we propose explicit recommendations for novel experimental designs. For MI tasks, paradigms ought to prioritize visually guided actions (e.g., mentally simulated reaching, grasping, or intercepting moving targets). These spatial tasks reliably recruit the frontoparietal networks that drive visuomotor coupling, making them more suitable than kinesthetic forms of imagery. For EM metrics, researchers should capture a comprehensive profile of oculomotor behavior, including pupil diameter and blink frequency, that serve as continuous indices of cognitive load and task engagement. Simultaneously, tracking fixations and smooth pursuit characteristics provides spatial information to verify the intended target of the MI command and reveals the underlying visuomotor strategy. Finally, saccadic kinematics could represent reliable indicators of user fatigue over the duration of the session. Combining complex MI tasks with comprehensive EM monitoring allows future studies to directly evaluate the proposed hybrid framework.

Analytical frameworks must also evolve with the aim to model the dynamic and non-linear interactions between EM and EEG. Linear correlations are likely insufficient to account for effects where eye movements might stabilize erratic neural signals. Moreover, a constraint in model training is the difficulty of accurate data labeling since internal states (e.g., fatigue) lack the precise timestamps accompanying discrete motor commands. Validation measures must expand to include quantitative measures of the inferred cognitive state itself because demonstrating a reduction in user frustration or cognitive load is essential to justify the integration of passive monitoring.

From a research perspective, rigorous evaluation inherently demands a longitudinal approach. Short-term studies frequently fail to detect how the coupling between EM and MI evolves throughout the BCI learning curve. Investigating learning effects and the long-term stability of passive EM integration necessitates studies that explore naturalistic environments. These factors influence BCI architecture and indicate a need for modular designs that include dedicated real-time cognitive state assessment components with the ultimate goal of successfully using passive EM integration.

### 6.3 Future research directions

Realizing the full value of integrating passive EM with active MI-BCIs depends on addressing several targeted objectives. Of primary importance is the systematic exploration of feature reliability. Thorough assessment is necessary to quantify the synergistic value of various passive EM features across diverse MI paradigms and user populations. Validation cannot stop at healthy volunteers and should encompass a more comprehensive range including populations affected by oculomotor deficits. This is a critical barrier as, depending on the specific deficit and stage of recovery, an estimated 7 to 86% of stroke patients exhibit oculomotor alterations (Rowe et al., 2009; Ciuffreda et al., 2007). Such high prevalence could potentially limit the immediate applicability of passive EM monitoring for a significant subset of the very population that most requires BCI-assisted motor rehabilitation. Hence, future research must verify whether passive EM features remain robust even in the presence of competing pathological eye signals (e.g., nystagmus or gaze palsies). In parallel, machine learning architectures for model fusion need to be tailored to capture the connection between passive EM and active EEG patterns under these clinical constraints.

Apart from the aforementioned clinical constraints, the technical challenge of signal non-stationarity should be directly addressed. While EEG data is notoriously non-stationary, it is important to acknowledge that ocular movements and pupillary diameters also fluctuate over time as users fatigue or modify their task strategies. However, integrating passive EM does not compound this issue, but it provides an alternative temporal perspective. Specific oculomotor metrics, which include pupillary responses or fixation distributions, can be evaluated over larger time bins, as they possess a relatively lower temporal resolution compared to raw EEG. This allows them to act as a more stable contextual baseline that varies relatively slowly. Future adaptive algorithms should specifically exploit this covariance by using the slower physiological drift of EM to adjust the decoding parameters of the highly non-stationary EEG signal.

As mentioned, progress also depends on transitioning from controlled laboratory settings to the real world. Longitudinal usability studies offer the only viable path to capture learning effects and evaluate system stability in key application areas like home-based neurorehabilitation. Taking a step further, proving functionality outside the lab means investigating these platforms using wearable technology. A clear justification exists for applying multimodal approaches to clarify the neural basis and comprehend the pathways that link specific EM patterns to fluctuations in cognitive states. Finally, the capacity to infer internal states raises ethical questions that transcend standard motor control issues. Because EM can reveal sensitive cognitive and emotional states, data privacy concerns become paramount. Thus, preemptively addressing concerns regarding user autonomy and data security should ensure that these systems remain responsible tools for both healthy and vulnerable populations.

## 7 Conclusion

This review has explored the multifaceted intersection of motor imagery (MI), eye movements (EM), and brain-computer interface technology (BCIs). Rather than following conventional methodologies, we centered our analysis on the underused potential of passive EM monitoring as a continuous indicator of cognitive state. This work seamlessly connects the foundational principles of motor cognition with the practical engineering constraints of BCI design. We argue that passive EM serve as more than secondary inputs and instead facilitate a transition toward inherently interactive and cognitively sensitive BCIs. By decoding subtle oculomotor cues regarding attention and cognitive load, these hybrid solutions gain the ability to adapt in real time fostering interactions that are dependable and less effortful. Although challenges regarding feature selection and individual variability remain pronounced, the prospect of BCIs that interpret and respond to internal user states holds extensive promise. Combining passive EM with active EEG-based control is a concrete step toward making BCI technology reliable enough for applied use.
